# Epigenetic Induction of Cancer-Testis Antigens and Endogenous Retroviruses at Single-Cell Level Enhances Immune Recognition and Response in Glioma

**DOI:** 10.1158/2767-9764.CRC-23-0566

**Published:** 2024-07-26

**Authors:** Thomas J. Lai, Lu Sun, Kevin Li, Terry J. Prins, Janet Treger, Tie Li, Matthew Z. Sun, David A. Nathanson, Linda M. Liau, Albert Lai, Robert M. Prins, Richard G. Everson

**Affiliations:** 1 Department of Neurosurgery, David Geffen School of Medicine, University of California, Los Angeles, Los Angeles, California.; 2 Department of Neurology, David Geffen School of Medicine, University of California, Los Angeles, Los Angeles, California.; 3 Department of Medical and Molecular Pharmacology, David Geffen School of Medicine, University of California, Los Angeles, Los Angeles, California.; 4 UCLA Neuro-Oncology Program, University of California, Los Angeles, Los Angeles, California.; 5 Jonsson Comprehensive Cancer Center, University of California, Los Angeles, Los Angeles, California.; 6 Parker Institute for Cancer Immunotherapy, San Francisco, California.

## Abstract

Glioblastoma (GBM) is the most common malignant primary brain tumor and remains incurable. Previous work has shown that systemic administration of Decitabine (DAC) induces sufficient expression of cancer-testis antigens (CTA) in GBM for targeting by adoptive T-cell therapy *in vivo.* However, the mechanisms by which DAC enhances immunogenicity in GBM remain to be elucidated. Using New York esophageal squamous cell carcinoma 1 (NY-ESO-1) as a representative inducible CTA, we demonstrate in patient tissue, immortalized glioma cells, and primary patient-derived gliomaspheres that basal CTA expression is restricted by promoter hypermethylation in gliomas. DAC treatment of glioma cells specifically inhibits DNA methylation silencing to render NY-ESO-1 and other CTA into inducible tumor antigens at single-cell resolution. Functionally, NY-ESO-1 T-cell receptor–engineered effector cell targeting of DAC-induced antigen in primary glioma cells promotes specific and polyfunctional T-cell cytokine profiles. In addition to induction of CTA, DAC concomitantly reactivates tumor-intrinsic human endogenous retroviruses, interferon response signatures, and MHC-I. Overall, we demonstrate that DAC induces targetable tumor antigen and enhances T-cell functionality against GBM, ultimately contributing to the improvement of targeted immune therapies in glioma.

**Significance:**

This study dissects the tumor-intrinsic epigenetic and transcriptional mechanisms underlying enhanced T-cell functionality targeting decitabine-induced cancer-testis antigens in glioma. Our findings demonstrate concomitant induction of tumor antigens, reactivation of human endogenous retroviruses, and stimulation of interferon signaling as a mechanistic rationale to epigenetically prime human gliomas to immunotherapeutic targeting.

## Introduction

Successful therapy for glioblastoma (GBM), the deadliest and most common malignant primary brain cancer, remains elusive. Immunotherapy is emerging as a promising treatment modality in many cancer sites, however multiple factors contribute to current limitations for use in the brain (1). Along with limited immune cell infiltration and an inhibitory immune microenvironment in GBM, the recognition of tumor-associated antigens by immune effector cells remains a major challenge (2, 3).

Endogenously expressed in many cancer types, cancer-testis antigens (CTA) are a family of proteins that are expressed normally in germinal spermatogonia and downregulated in somatic tissue via *de novo* DNA methylation and other epigenetic programs (4). Often aberrantly expressed in tumor tissue, New York esophageal squamous cell carcinoma 1 (NY-ESO-1) and other well-studied CTA are currently being assessed in a variety of adoptive T cells and cancer vaccine clinical trials in numerous cancer types (5–7).

However, despite the potential for tumor specificity and epitope immunogenicity, current approaches targeting CTA have not been successful to date. Single-antigen therapies are susceptible to antigen escape and engineered T cells struggle to overcome inhibitory tumor-immune microenvironments within solid tumors (8). Moreover, in immunogenically cold tumors like GBM, NY-ESO-1 and other CTA are not frequently endogenously expressed (9).

Previous work from our group and others have shown that clinically achievable doses of the FDA-approved DNA methyltransferase inhibitor (DNMTi), Decitabine (DAC), induces expression of CTA in GBM tumor tissue but not normal tissue for immunotherapeutic targeting (10–13). Specifically, our group has published preclinical proof of principle that DAC induces NY-ESO-1 expression in intracranial human glioma xenografts for adoptive T-cell infiltration and targeting *in vivo* (14). However, the exact mechanisms by which DAC enhances NY-ESO-1 adoptive T-cell therapy in GBM remain to be elucidated. In particular, the re-expression of immunogenic targets silenced by DNA methylation in GBM patient tumors has not been profiled at a single-cell resolution. Moreover, the tumor-intrinsic DAC-induced epigenetic and transcriptional changes that contribute to enhanced immunogenicity beyond antigen expression in GBM remain to be delineated. We hypothesized that DAC-induced gene expression programs would lead to the immunosensitization of GBM via the concomitant re-expression of multiple tumor-specific CTA targets and enhance the immunotherapeutic milieu through reactivation of human endogenous retroviruses (hERV) and the pursuant antiviral-like interferon response.

To this end, we determine that DNA hypermethylation is associated with low expression of NY-ESO-1 and other CTA in serum-free primary patient-derived gliomasphere lines, immortalized glioma cell lines, and GBM patient tissue samples. We demonstrate that DAC induces NY-ESO-1 expression and targeting in GBM through the reversal of DNA methylation. Targeting of DAC-treated glioma cells stimulates polyfunctional NY-ESO-1 CD8^+^ responses for specific and robust antitumor clearance. DAC upregulates other CTA across diverse gliomasphere single-cell populations concomitantly with reactivation of hERV and enhanced interferon signaling in primary gliomaspheres.

## Methods

### Cell lines and biological samples

A total of 27 tissue specimens were obtained from the UCLA Brain Tumor Translational Resource. Remnant human brain tumor samples were collected from patients undergoing surgical resection and who provided written informed consent. The collection of human brain tumor samples was approved by the UCLA Institutional Review Board. *IDH1* was sequenced on all samples used for NY-ESO-1 methylation studies.

The human GBM cell line U-251MG (RRID:CVCL_0021) was obtained from the American Type Culture Collection and authenticated by short tandem repeat. Primary human GBM cell line DBTRG-05MG (RRID:CVCL_1169) and human meningioma cell line CH-157MN (RRID:CVCL_5723) were obtained via material transfer agreement from Dr. Carol Kruse (Department of Neurosurgery, UCLA) and Dr. G. Yancey Gillespie (Department of Neurosurgery, University of Alabama at Birmingham), respectively. The human primary gliomaspheres GS277, GS025, GS081, GS243, and GS351 were supplied by Dr. D.A. Nathanson (Department of Molecular and Medical Pharmacology, UCLA) and the human primary gliomaspheres HK211, HK213, HK308, and HK252 were supplied by Dr. Harley Kornblum (Department of Molecular and Medical Pharmacology, UCLA). Cells were not routinely mycoplasma tested. HLA-A^∗^0201 haplotype was confirmed via DNA sequencing in U-251MG, GS277, and DBTRG-05MG.

Peripheral blood mononuclear cells (PBMC) were obtained from healthy human volunteers at UCLA Medical Center after leukapheresis and isolated by density gradient centrifugation. Written informed consent and institutional review board approval were obtained for all studies involving human blood and tissues.

### *In vitro* immortalized and primary gliomasphere cell treatment with DAC

DAC (Cayman Chemical), was reconstituted in dimethyl sulfoxide (DMSO) as a 20 mmol/L stock solution. U-251MG, CH-157MN, and DBTRG-05MG cells were cultured overnight (day 0) at 1.5 × 10^6^ per 10 cm dish in Dulbecco’s Modified Eagles Medium (DMEM)/F12 + 10% Fetal Bovine Serum + 1% Penicillin/Streptomycin (Invitrogen). Media was replaced starting at day 1 with fresh media supplemented with 1-µmol/L DAC every day for 4 days. Cells were split and replated at 1.5 × 10^6^ on the day 2 and day 4 of treatment cycle and harvested at day 7.

Primary gliomaspheres were seeded overnight (day 0) at a cell density of 2.0 × 10^6^ cell in 20 mL serum-free DMEM/F12 + 2% B27 supplement without Vitamin A + 1% Glutamax + 1% penicillin-streptomycin media (Invitrogen). Heparin (Sigma), human epidermal growth factor, and human fibroblast growth factor were supplemented into growth media every 5 days at final concentrations of 5 µg/mL, 50 ng/mL, and 20 ng/mL, respectively (Gibco). DAC was added starting at day 1 at a final concentration of 0.5 µmol/L for 4 days. Treatment cycle was repeated for 3 weeks and harvested at day 7 time point.

### HLA*A0201-transduced CH-157MN (CH157-A2)

The murine stem cell virus (MSCV) based lentiviral vector encoding HLA-A*0201 was obtained from Dr. Antoni Ribas (Department of Medicine, UCLA). A total of 1 × 10^5^ CH-157MN cells were plated overnight in a 6-well plate. Culture medium was aspirated the following day before viral supernatant and protamine sulfate (1 mg/mL) were added and cultured overnight. The following day, the cells were washed with phosphate-buffered saline (PBS) and were passaged as needed. When labeled with APC-BB7.2 antibody (anti-HLA-A*0201; RRID:AB_10646036) and assayed via flow cytometry, >98% were HLA-A*0201 positive.

### NY-ESO-1 T-cell receptor-transduced lymphocytes

The PG13-based stable retroviral packaging cell line encoding an HLA-A*0201—restricted NY-ESO157—165 specific T-cell receptors (TCR) were generated as described and obtained from Dr. Paul Robbins (Surgery Branch, NCI/NIH; ref. 15). Anti-CD3 (clone OKT-3; 50 ng/mL; RRID:AB_467057) was used to stimulate human PBMCs for 48 hours prior to transduction with supernatants from the PG13-based retroviral producer cell line, as described. The transduced PBMC were expanded for 3 days in the presence of 300 IU interleukin (IL)-2 and then rested for 1 day in 30 IU IL-2. When labeled with FITC-vB13.1 antibody (anti-NY-ESO-1 TCR; RRID:AB_2564031) and assayed via flow cytometry, >75% of CD8 positive cells were NY-ESO-1 TCR positive (Supplementary Fig. S2A).

### *In vitro* cytotoxicity assays

Cytotoxic killing of tumor cells was assessed using the xCELLigence Real-Time Cell Analyzer System (Agilent). HLA-A*0201 Target cells (CH157-A2 and U-251MG DAC-treated or DMSO vehicle) were plated at day 0 (15,000 cells/well) in 100 µL of medium. After overnight tumor cell adherence to the well bottom, effector (NY-ESO-1 TCR transduced T cells) and mock effector (untransduced PBMC) were added at effector:target (E:T) ratios of 1:10, 1:5, 1:2, and 1:1. Cell index values (relative cell impedance) were collected over 40 hours and normalized to the maximal cell index value 1 hour after effector cell plating. The percentage lysis at a time point was calculated as a proportion of the normalized cell index of target cells plated with effector cells versus the normalized cell index of target cells plated with mock effector cells.

For experiments with primary gliomaspheres, target HLA-A*0201 positive gliomaspheres (GS277 DAC or DMSO) were plated at day 0 (15,000 cells/well, 100 µL medium) in laminin-pretreated wells (Gibco). After overnight tumor cell adherence to the well bottom, effector (NY-ESO-1 TCR transduced T cells) and mock effector (untransduced PBMC) were added at E:T ratios of 5:1, 2:1, 1:1, and 1:2. Normalized cell index and percentage specific lysis were determined as above.

### *In vitro* isoplexis single-cell secretomics co-culture assay

CD8 positive (CD8^+^) T cells were separated from NY-ESO-1 TCR–transduced PBMC or untransduced PBMC via CD8^+^ T cell Isolation Kit (Miltenyi Biotech). CD8^+^ cells were co-cultured with DAC treated or untreated DBTRG-05MG for 24 hours at 1:2 E:T ratio. Stimulated CD8^+^ cells were collected from the co-culture and loaded into IsoLight fluorescent flow cell (Isoplexis) containing assay panel of 32 key immunologically relevant molecules. Individual cell and cytokine profile measured as described by Rossi and colleagues (16). Percent polyfunctional cells was determined as number of cells secreting 2+ cytokines divided by total number of cells in sample. Polyfunctional strength index (PSI) was determined as mean fluorescence intensity (MFI) per sample multiplied by number of polyfunctional cells in sample. Isolight single-cell secretomics assay was performed by Isoplexis. Fluorescence values per cytokine per cell are included in the supplemental data (Supplemental Data S1).

### Bisulfite sequencing and TA cloning

Genomic DNA was isolated from tumor samples (Qiagen) and prepared with sodium bisulfite using the EZ Methylation Gold Kit (Zymo Research). Nested PCR primers were designed to amplify the CpG island sequence. Sequences were amplified using Platinum II Taq polymerase (Invitrogen), before the PCR product was purified (Qiagen) and sequenced according to BigDye Terminator v3.1 chemistry (Applied Biosystems).

PCR sequences were reviewed using Chromas Lite 2.33 (Technelysium Pty Ltd.), and CpG sites exhibiting a Cytosine to Thymine (C/T) ratio <0.4 were considered demethylated, 0.4> C/T >0.6 considered partially demethylated, and C/T >0.6 considered methylated.

For quantification of *CTAG1A/B* promoter methylation levels after DAC exposure, the T-A cloning method was applied. Following bisulfite conversion and PCR, the amplified *CTAG1A/B* CpG island products were ligated into pGEM-Teasy vector (Promega). Ligated products were identified by blue-white screening and amplified by miniprep (Invitrogen). The resulting *CTAG1A/B* fragment within *CTAG1A/B*-pGEM-Teasy plasmids were further amplified by PCR using T7/Sp6 primer pair and sequenced with sequenced according to BigDye Terminator v3.1 chemistry with T7 primer.

### Primer preparation for bisulfite PCR

We designed two pairs of forward and reverse primers to perform two nested PCR of our bisulfite converted DNA. CpG islands were determined by the MethPrimer online program and primers were designed to avoid CpG sites (Supplementary Fig. S1C; ref. 17). The F1/R2 primer pair was used for the first PCR and the F1/R1 primer pair for the second PCR. The F3 primer was used for BigDye sequencing.

• *CTAG1A/B* F1: 5′-TTAGGAGGTTTTGGTATTTTTGATG
• *CTAG1A/B* R1: 5′-CTACAACATCCATTCAACCCTAAA
• *CTAG1A/B* R2: 5′-TCAAAACAAAACAAAACAAAACAAA
• *CTAG1A/B* F3: 5′ATGGTTTAGGGGGTAATGTTGG

### Quantitative real-time PCR

Total RNA was isolated using the RNeasy Mini Kit (Qiagen) according to the manufacturer’s protocol. Quantitative real-time qPCR was performed using the LightCycler RT-qPCR system with the LightCycler SYBR green mastermix (KAPA Biosystems). *GAPDH* was used as the housekeeping gene for expression normalization. Primer sequences were as follows:

• *CTAG1B* Forward: 5′-TGT​CCG​GCA​ACA​TAC​TGA​CT
• *CTAG1B* Reverse: 5′-ACT​GCG​TAG​TCC​ACA​TCA​AC
• *GAPDH* Forward: 5′-CGC​CCA​ATA​CGA​CCA​AAT​C
• *GAPDH* Reverse: 5′-AGC​CAC​ATC​GCT​CAC​ACA​C

For hERV RT-qPCR, the following primer sequences were used according to Strissel and colleagues (18):

• *ERV3* Forward: 5′-AAC​TAA​TGC​CCC​AAG​ATA​ATT​TCA
• *ERV3* Reverse: 5′-TTA​AGA​ACC​AGA​TGC​TCT​GAC​TTG
• *ERW1*/Syncytin-1 Forward: 5′-ATG​GAG​CCC​AAG​ATG​CAG
• *ERW1*/Syncytin-1 Reverse: 5′-AGATCGTGGGCTAGCAG

### Single-cell RNA sequencing

DAC- and vehicle-treated GS025 and GS243 cells were suspended in 1× PBS + 0.04% bovine serum albumin at a concentration of 1,000 cells/μL after passage. Single-cell libraries were prepared according to 10× genomics 3′v3.1 20× library preparation (10× Genomics). Libraries were run on one lane at single-end, 1 × 75 base pair (bp) at 200 million reads/sample and 40 k reads/cell for paired end sequencing (Illumina). Single-cell library construction and sequencing was performed by the UCLA Technology Center for Genomics and Bioinformatics Core Laboratory (TCGB).

NY-ESO-1 protein is encoded by two genes, *CTAG1A* and *CTAG1B*, which are identical in sequence but run in opposite directions. To effectively map *CTAG1B* reads to the genome, raw data were aligned using Cell Ranger (10× Genomics), version 3.0 or higher, against a custom GrCH38 human genome omitting the identical *CTAG1A* gene. Mapping to the full genome led to multiple mapping of *CTAG1A* and *CTAG1B* reads, resulting in the Cell Ranger algorithm discarding all NY-ESO-1 reads. Aligned data were integrated and analyzed with Seurat (RRID:SCR_016341) R package, version 4.2.0 (19). Only cells containing more than 200 features and less than 20% mitochondrial or 40% ribosomal features were included in analysis as described (20). The raw transcript count for each sample was individually normalized using the NormalizeData function before being merged and integrated using anchor-based reciprocal principal component analysis using the IntegrateData function. Different cell cluster populations were defined using the FindNeighbors function and the genes that were differentially expressed in each cluster or treatment were computed using the FindMarker or FindAllMarker function. Single-cell–level gene-set enrichment was computed using Seurat’s AddModuleScore function.

### DNA extraction for probe-based methylation array

Genomic DNA and total RNA were isolated using Quick DNA/RNA Miniprep Kit according to manufacturer’s protocol (Zymo Research).

Genomic DNA (50 ng) was bisulfite converted using the EZ Methylation Gold Kit (Zymo Research). Bisulfite-converted DNA was hybridized to Human MethylationEPIC BeadChip (Illumina) probes specific for methylation status at genomic locus. The hybridized chip was scanned using Illumina iScan and scans were analyzed in R using the minfi (RRID:SCR_012830) package, version 1.44.0, and “IlluminaHumanMethylationEPICanno.ilm10b4.hg19” annotation file (21). Methylation beta value was determined as the ratio of fluorescence intensity between methylated (M) and unmethylated (U) probes at an interrogated CpG locus [M/(M + U)]. Probe hybridization and scanning was performed by the UCLA Neurogenomics Sequencing Core Laboratory.

### Bulk RNA sequencing

Paired-end, 2 × 100 bp transcriptome reads were mapped to the Genome Reference Consortium Human Build 38 (GRCh38) reference genome using HISAT2 (RRID:SCR_015530), version 2.2.1, and PCR duplicates were removed with RmDup in Picard (RRID:SCR_006525), version 2.25.0 (22). The gene level counts were generated by HTSeq-count (RRID:SCR_011867), version 2.0.2; we took log_2_ counts per million as normalized gene expression values (23). Differential expression analysis between DAC treated and control was performed using DESeq2 (RRID:SCR_000154) R package, version 1.38.3 (24). For gene set enrichment analysis, the Enricher function in clusterProfiler (RRID:SCR_016884) R package, version 4.6.2 (25), was used to generate *P*-values of the Hallmark gene sets from the Broad Institute’s Molecular Signatures Database (RRID:SCR_016863; ref. 26).

hERV counts were determined as outlined by Engel and colleagues (27). Briefly, raw transcriptome reads were uploaded to the Galaxy platform and aligned to a synthetic virus metagenome using default settings in Bowtie2 (Galaxy Version 2.5.0+galaxy0; RRID:SCR_016368). PCR duplicates were removed using RmDup (Galaxy Version 2.0.1). The gene level counts were generated by featureCounts (Galaxy Version 2.0.3+galaxy2; RRID:SCR_012919) according to the following parameters: paired end reads counted as one single fragment, only allow fragments with both reads aligned (-B), reads mapping to multiple regions were excluded (-O, -M, and -fraction). hERV family counts were quantified via fragments per kilobase million (FPKM) using the approximate library size when aligned to (GRCh38) reference genome. hERV gene family FPKM were further normalized to the FPKM of housekeeping gene family containing sequences for hypoxanthine phosphoribosyl transferase 1 (*HPRT1*), glyceraldehyde-3-phosphate dehydrogenase (*GAPDH*), and ubiquitin B (*UBB*) within the viral metagenome. Raw counts and FPKM values have been included in the supplemental data (Supplemental Data S2).

Library construction and sequencing was performed by the UCLA TCGB.

### Schematic figures

Isoplexis experimental design and immune panel schematics (Supplementary Fig. S3A and S3B) were generated with Biorender.com.

### Statistical analysis

Statistical tests for each figure are indicated in the figure legend. Continuous variables were compared using Student *t* test. Results comparing more than two groups were analyzed by ANOVA followed by Kruskal–Wallis statistics. Other than the differentially expressed genes called by Seurat, DESeq2, and clusterProfiler, all *P* values were nominal (unadjusted) *P* values and calculated using GraphPad Prism (RRID:SCR_000306). A *P* value less than or equal to 0.05 was considered significant.

### Data availability

The sequencing and microarray data generated in this study are publicly available in Gene Expression Omnibus accession GSE261191 (RRID:SCR_005012).

## Results

### CpG island hypermethylation correlates with low endogenous expression of NY-ESO-1 in GBM and reduced NY-ESO-1–specific T-cell cytolysis

To evaluate the prevalence of endogenous CTA expression in primary brain tumors, we accessed The Cancer Genome Atlas for GBM (TCGA_GBM) using “TCGAbiolinks” R package, version 2.25.3 (28). We confirmed that the mRNA expression of a list of previously published CTA (29), including *CTAG1A/B* that encode for NY-ESO-1 antigen, were downregulated in the majority of the 153 TCGA_GBM samples. We further subset the TCGA_GBM dataset into 11 isocitrate dehydrogenase (*IDH*) mutant (IDHmut) and 142 IDH wild-type (IDHwt) gliomas to further evaluate CTA expression in correlation with this well-characterized prognostic glioma biomarker. Despite IDHmut GBM canonically exhibiting differential epigenetic/methylation profiles compared to IDHwt (30), TCGA_GBM demonstrated neither significant differential expression of CTA nor NY-ESO-1 between IDHwt and IDHmut or IDHwt and healthy brain tissue (Supplementary Fig. S1A). Similar stratification of TCGA_GBM on *MGMT* promoter methylation status—another well-characterized epigenetic biomarker for glioma chemosensitivity—demonstrated neither significant differential NY-ESO-1 nor CTA expression (Supplementary Fig. S1B; ref. 31).

**Figure 1:**
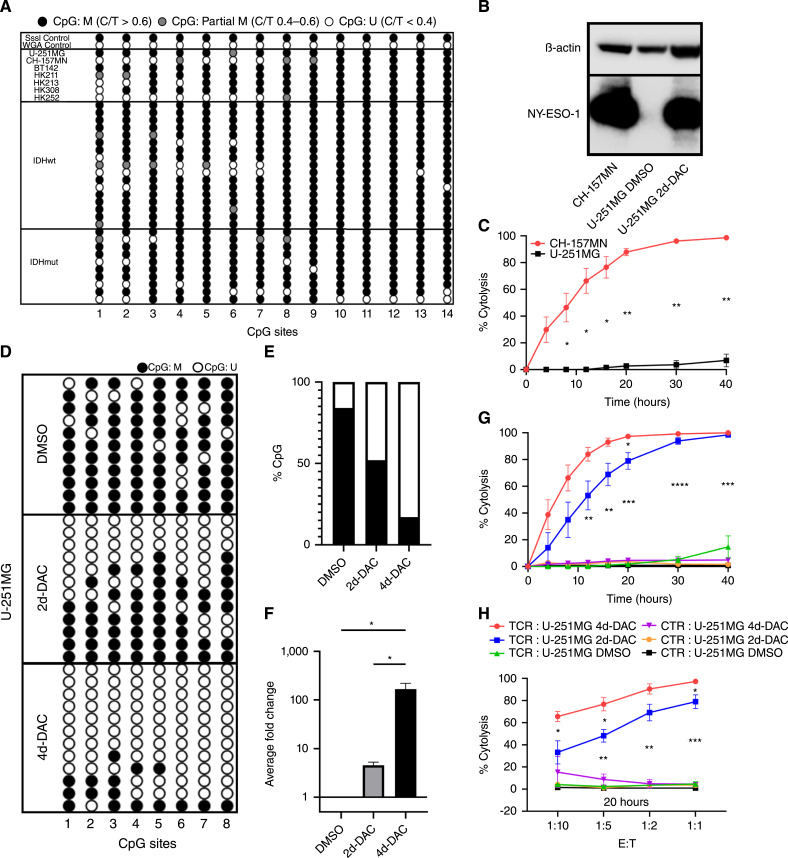
CpG island methylation is associated with *CTAG1A/B* downregulation in the majority of gliomas and DAC time-dependently induces NY-ESO-1 expression via promoter demethylation for targeting by NY-ESO-1-specific TCR*.***A,** Representative lollipop diagram of direct methylation PCR sequencing of immortalized GBM cell lines (U-251MG, BT142), immortalized meningioma cell line (CH-157MN), primary GBM cell lines (HK211, HK213, HK308, HK252), IDHwt (*n* = 17) and IDHmut (*n* = 10) patient samples. M, Methylated and U, Unmethylated determined from cytosine/thymine (C/T) peak ratio. **B,** Representative Western blot of native CH-157MN and U-251MG and U-251MG treated with 1 µmol/L DAC for 2 days. **C,** Cytotoxic analysis of U-251MG and CH-157MN cells over a 40-hour period when co-incubated with NY-ESO-1 TCR-transduced effector T cells (*n* = 2; **, *P* < 0.01; ***, *P* < 0.001; ****, *P* < 0.0001; unpaired *t* test). **D,** Representative Bis-Seq-TA cloning lollipop diagram and (**E**) percent methylated CpGs to total CpGs of first eight sites of analyzed CpG island in U-251MG cells treated with DMSO or 1-µmol/L DAC for 2 or 4 days. **F,** Average fold change of mRNA expression of treated U-251MG (*n* = 3; *, *P* < 0.05; one-way ANOVA). **G,** Cytotoxic analysis of treated U-251MG cells over a 40-hour period when co-incubated with NY-ESO-1 TCR-transduced effector T cells at E:T ratio of 1:1 and (**H**) at multiple E:T ratios (1:10, 1:5, 1:2, 1:1) at 20-hour (*n* = 3; *, *P* < 0.05; **, *P* < 0.01; ***, *P* < 0.001; ****, *P* < 0.0001; multiple unpaired *t* tests).

*CTAG1A* and *CTAG1B* are exact in sequence but are located only 30,000 base pairs apart on the X-chromosome (UCSC Genome Browser). To assess the DNA methylation regulation of NY-ESO-1 expression in GBM, we analyzed a 179 base pair CpG island containing 14 CpG sites beginning 125 base pairs downstream from the transcription start site (Supplementary Fig. S1C; ref. 12). We analyzed the methylation profile using bisulfite converted genomic DNA from 27 patient tumors, four primary gliomasphere lines, and three immortalized cell lines. As expected, direct PCR bisulfite Sanger sequencing methylation analysis of these samples identified hypermethylation in this region. Subsetting of patient tumor samples into 10 IDHmut or 17 IDHwt tumors revealed no significant difference in methylation profile consistent with TCGA_GBM database mRNA expression findings (Fig. 1A).

In contrast to gliomas, meningiomas have previously been reported to endogenously express high levels of NY-ESO-1 (32, 33). Despite the difference in pathology, we hypothesized that DNA hypomethylation might also be associated with high basal NY-ESO-1 expression in meningiomas. As expected, we observed that the immortalized GBM cell line, U-251MG, exhibited hypermethylation while the immortalized meningioma cell line CH-157MN exhibited hypomethylation in the first eight CpG sites in this region (Fig. 1A). Compared to U-251MG, CH-157MN exhibited 1,000-fold greater *CTAG1B* mRNA transcript (Supplementary Fig. S1D). Similarly, Western blot of NY-ESO-1 protein level expression was inversely correlated with CpG island methylation status in these two cell lines (Fig. 1B). These results indicate that the *CTAG1A/B* CpG island methylation status is associated with the differential expression of NY-ESO-1 mRNA and protein between U-251MG and in CH-157MN.

To assess NY-ESO-1-specific targeting of endogenously expressed antigen in CH-157MN, we used an impedance-based xCelligence real-time killing assay. When co-cultured at an effector to target (E:T) ratio of 1:1 with >75% transduced NY-ESO-1 TCR engineered peripheral blood mononuclear cells (PBMC), HLA-A*0201 matched U-251MG and CH-157MN exhibited live cell adherence (measured as cell index) that positively correlated with their CpG island methylation profiles beginning at 10 hours after the addition of NY-ESO-1 TCR–specific PBMCs (TCR-T; Supplementary Fig. S2A and S2B).

We calculated for NY-ESO-1 TCR T-cell–specific cytolysis using the difference in normalized target cell adherence between our NY-ESO-1 TCR-T and donor-matched untransduced PBMC negative controls. Consistent with the normalized cell index after addition of TCR-T, CH-157MN 50% specific cytolysis (KT50) was reached at 11 hours following the addition of PBMC while U-251MG demonstrated negligible specific cytolysis even after 40 hours (Fig. 1C). Taken together, these data demonstrate that the methylation profile of the *CTAG1A/B* CpG Island is associated with NY-ESO-1 antigen expression and targeted cytolysis by NY-ESO-1 TCR-T.

### Decitabine reverses methylation silencing and induces robust upregulation of NY-ESO-1 in primary and immortalized glioma lines that is sufficient for targeting by antigen-specific T cells *in vitro*

We assessed the pharmacological mechanism of DAC to induce NY-ESO expression in glioma via the reversal of this hypermethylation profile in the *CTAG1A/B* CpG Island. In previous *in vivo* experiments, a clinically achievable DAC dosing strategy followed by NY-ESO-1 TCR-T adoptive transfer resulted in effective T-cell infiltration, tumor clearance, and extension of survival (14). Indeed, other preclinical and clinical studies have established that 1 µmol/L is a conservative concentration of DAC after penetration past the blood brain barrier (34, 35). Furthermore, previous preclinical studies have demonstrated that DAC-induced NY-ESO-1 expression remains durably upregulated through 65 days after removal of the drug in U-251MG (14). We therefore replicated this treatment scheme *in vitro* and validated that NY-ESO-1 protein is upregulated in U-251MG after low dose exposure to DAC (Fig. 1B).

Consistent with the *CTAG1A/B* methylation profile in CH-157MN, we noted that the majority of demethylation after DAC treatment occurred in the first eight CpG dinucleotides and we therefore focused our analysis on these sites (Supplementary Fig. S2C). Bisulfite TA cloning revealed that prolonged exposure to DAC time-dependently demethylated the CpG island of U-251MG (Fig. 1D). Two days of treatment produced a 32% reduction of CpG methylation within these eight CpG sites. After 4 days of treatment, these CpG sites demonstrated near-complete reversal of hypermethylation with a nearly 70% reduction in methylated CpG sites (Fig. 1E).

The time-dependent reversal of DNA methylation corresponded to *CTAG1B* mRNA induction. U-251MG treated with DAC for 2 days induced *CTAG1B* mRNA expression 4.6-fold compared to the DMSO vehicle control while 4 days of treatment induced a 166-fold increase compared to vehicle control (Fig. 1F).

Functionally, the time-dependency of DAC-induced *CTAG1A/B* CpG demethylation and subsequent antigen expression correlated with differences in efficiency of NY-ESO-1 TCR-T targeted cytolysis. At an E:T ratio of 1:1, target U-251MG cells exposed to DAC for 4 days exhibited a KT50 at 8 hours after introduction of NY-ESO-1 TCR-T while target cells treated for 2 days exhibited a KT50 at 12 hours after introduction of PBMC. Co-cultures containing DMSO vehicle controls with NY-ESO-1 TCR-T cells or DAC-treated target cells with untransduced PBMC controls exhibited negligible targeted cytolysis 40 hours after addition of T cells (Fig. 1G). This temporal difference between 2 and 4 days DAC exposure was consistent at 20 hours across a variety of E:T ratios (1:10, 1:5, 1:2, 1:1) with effective killing in both DAC treatment time points exhibited at an E:T ratio of 1:5 (Fig. 1H) Altogether, these results demonstrate that DAC reverses *CTAG1A/B* CpG hypermethylation silencing to induce NY-ESO-1 antigen upregulation for targeting by NY-ESO-1 TCR-T PBMC.

### Targeting of DAC-immunosensitized primary GBM cells promotes specific, robust, and polyfunctional immune responses in CD8^+^ NY-ESO-1 TCR-T

**Figure 2:**
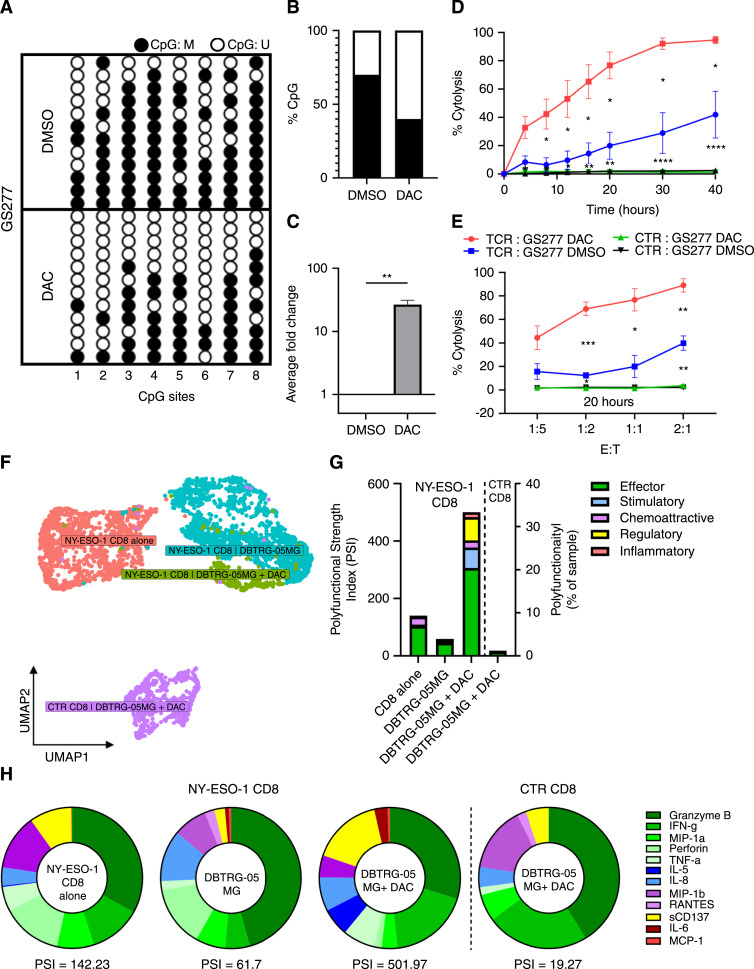
DAC immunesensitization promotes specific, robust, and polyfunctional NY-ESO-1 TCR CD8^+^ T-cell responses in primary GBM cell lines. **A,** Representative Bis-Seq-TA cloning lollipop diagram and (**B**) percent of methylated CpGs to total CpGs of first eight sites of analyzed CpG island in primary GS277 cells treated with DMSO or 0.5 µmol/L DAC for 12 days. **C,** Average fold change of mRNA expression of treated GS277 (*n* = 3; **, *P* < 0.01; two-tailed unpaired *t* test). **D,** Cytotoxic analysis of treated GS277 over a 40-hour period when co-incubated with NY-ESO-1 TCR-transduced effector T cells at E:T ratio of 1:1 and (**E**) at multiple E:T ratios (1:5, 1:2, 1:1, 2:1) at 20-hour (*n* = 3; *, *P* < 0.05; **, *P* < 0.01; ***, *P* < 0.001; ****, *P* < 0.0001; multiple unpaired *t* tests). **F,** Uniform manifold approximation projection (UMAP) of CD8^+^ T cells transduced with NY-ESO-1 TCR CD8 or untransduced control CD8 (CTR) from four stimulated groups: NY-ESO-1 CD8^+^ alone (*n* = 1665 CD8^+^ cells), NY-ESO-1 CD8^+^ stimulated with DBTRG-05 (*n* = 1902 CD8^+^ cells), NY-ESO-1 CD8^+^ stimulated with DBTRG-05 + DAC (*n* = 284 CD8^+^ cells), CTR CD8 stimulated with DBTRG-05 + DAC (*n* = 807 CD8^+^ cells). **G** and **H,** PSI defined as the percentage of polyfunctional cells, multiplied by MFI of the proteins secreted by the polyfunctional cells for stimulated CD8^+^ at the single-cell resolution across DBTRG-05 co-cultures.

We next evaluated the induction and specific targeting of NY-ESO-1 via DAC-induced CpG demethylation in a serum-free patient-derived HLA*A0201 positive gliomasphere line, GS277. GS277 were harvested after 3 weeks of exposure to a final concentration of 0.5 µmol/L DAC (36). Similar to 1 µmol/L 2d-DAC in U-251MG the *CTAG1A/B* CpG island exhibited reversal of its hypermethylation profile that likewise resulted in a 30% reduction in methylated CpG sites and a 23-fold increase in *CTAG1B* mRNA expression (Fig. 2A–C). Consistent with U-251MG, NY-ESO-1 induction in primary GS277 resulted in increased targeted cytolysis compared to DMSO vehicle controls. Within 40 hours following the addition of NY-ESO-1 TCR-T, DAC-treated GS277 achieved KT50 around 12 hours at an E:T of 1:1 (Fig. 2D). Furthermore, assessing a range of E:T ratios (1:5, 1:2, 1:1, 2:1) at 20 hours post T cells addition, we observed specific killing of DAC-treated primary GS277 at an E:T of 1:2 (Fig. 2E).

To better characterize how DAC functionally immunosensitizes primary glioma cells to recognition by NY-ESO-1 TCR engineered CD8^+^ T cells, we co-cultured NY-ESO-1 TCR-T with primary DBTRG-05MG GBM cells treated with or without DAC. After 24 hours, T cells were isolated from the co-culture and assayed for the secretion of a panel of 32 immunologically relevant molecules using the Isolight single-cell secretomics platform (Isoplexis; Supplementary Fig. S3A and S3B).

Polyfunctional diversity has been a previous indicator of patient response in chimeric antigen receptor engineered T cells (CAR-T) targeting CD19 in non–Hodgkin lymphoma (16). Compared to 20% to 25% in CD19 CAR-T, over 30% of NY-ESO-1 CD8^+^ TCR-T cells showed marked polyfunctional upregulation by the stimulation of DBTRG-05MG target cells pretreated with DAC compared to target cells without DAC (Supplementary Fig. S3C).

After dimensional reduction, NY-ESO-1 CD8^+^ TCR-T co-cultured with DAC-treated DBTRG-05MG resulted in a distinct secretomic profile (Fig. 2F). CD8^+^ NY-ESO-1 TCR-T cells demonstrated an elevated PSI greater than 500 (Fig. 2G). In previous studies, equivalent PSI, defined as the percentage of polyfunctional cells multiplied by MFI of the proteins secreted by the polyfunctional cells, corresponded to enhanced clinical responses in CD19 CAR-T (16). As expected, NY-ESO-1 CD8^+^ TCR-T stimulated with untreated DBTRG-05MG and untransduced CD8^+^ (CTR CD8) stimulated with DAC-treated DBTRG-05MG resulted in limited recognition and low polyfunctional cytokine secretion.

The polyfunctional profile of the CD8^+^ NY-ESO-1 TCR-T cells stimulated with DAC-treated DBTRG-05MG were primarily composed of effector proteins associated with antitumor, antiviral, and T helper cell cytokine repertoire. Granzyme B, IFN-g, sCD137 followed by IL-5, IL-8, TNF-a, MIP-1a, MIP-1b, and perforin were major drivers of the markedly enhanced PSI and effector function of CD8^+^ NY-ESO-1 TCR-T cells against DBTRG-05MG target cells treated with DAC (Fig. 2H). Taken together, NY-ESO-1 specific TCR-T cells targeting DAC-treated GBM cells polyfunctionally secrete effector and immune-signaling factors that contribute to effective clearance of NY-ESO-1–expressing tumor cells.

### Despite glioma interpatient and intratumoral heterogeneity, DAC induces CTA homogeneously in serum-free patient-derived gliomaspheres with enhanced antigen presentation

To evaluate the tumor-intrinsic transcriptional changes resulting from DAC treatment that may contribute to this enhanced T-cell activity, we performed single-cell RNA sequencing (scRNA-seq) on DAC-treated patient-derived gliomaspheres, GS025 and GS243. Previous molecular characterization of these gliomaspheres defined GS025 as a classical GBM subtype and GS243 as a mesenchymal GBM subtype (37). After validating that DAC treatment resulted in reversal of *CTAG1A/B* CpG island hypermethylation in these two cell lines (Supplementary Fig. S4A), we integrated the datasets using reciprocal principal component analysis and analyzed the transcriptome from 21,322 individual cells across two DAC-treated (*n* = 6,115 in GS025_DAC; *n* = 2,505 in GS243_DAC) and two untreated samples (*n* = 7,258 in GS025_DMSO; *n* = 5,444 in GS243_DMSO).

**Figure 3:**
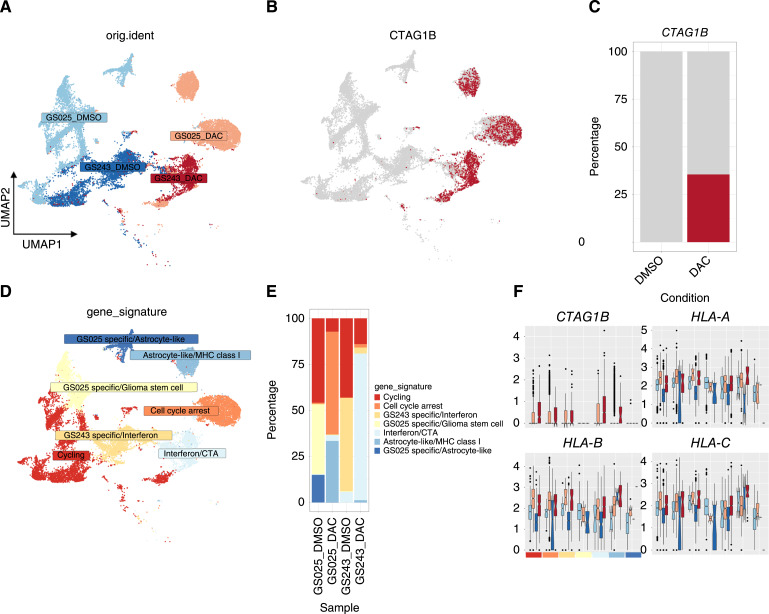
DAC induces NY-ESO-1 and other CTA homogeneously in serum-free patient derived gliomaspheres. **A,** UMAP of primary gliomasphere lines (GS025, GS243) treated with DAC or DMSO (*n* = 22,313; **B**) UMAP of cells expressing *CTAG1B* (red). **C,** 35% (*n* = 1,487 *CTAG1B*^+^ cells of 4,190 DAC-treated cells) of treated cells demonstrate *CTAG1B* upregulation. **D,** UMAP and (**E**) proportion of cell populations as determined by Seurat and differentially expressed cluster markers. **F,** Relative gene expression of cells expressing *CTAG1B* and pan-MHC class 1 (*HLA-A*, *HLA-B*, *HLA-C*) in GS025_DMSO (light blue), GS025_DAC (light red), GS243_DMSO (dark blue), and GS243_DAC (dark red) across defined cell populations.

To characterize the changes in cellular programs resulting from DAC, we performed dimensional reduction that demonstrated distinct transcriptional differences between DAC and vehicle controls (Fig. 3A). After removal of low-read cells, the clustering of patient-derived gliomasphere lines identified seven tumor cell subpopulations. Three of the seven clusters corresponded to DAC-treated populations and *CTAG1B* reads were detected across all three DAC-response clusters in 35.5% of DAC-treated cells (compared to 0% in vehicle treated cells; Fig. 3B and C).

The seven clusters were further characterized based on differentially expressed gene markers. Gliomasphere line specific (*EGFR*) and cell cycle markers (*MKI67*, *TOP2A*) dominated clustering and we noted enrichment of glioma stem cell (*PTPRZ1*, *SOX2*) and astrocytic markers (*GFAP*, *S100B*) in GS025. GS243 demonstrated enrichment of downstream interferon markers (*MX1*, *ISG15*). Within three DAC-response clusters, we noted a GS025 astrocyte-like cluster with enhanced MHC class I markers (*HLA-A/B/C*), a GS025 population undergoing cell cycle arrest (*CDKN1A*, *BTG2*), and a GS243 downstream interferon population with enhanced CTA expression (*ISG15*, *IFI27*, *PAGE2B*; Fig. 3D and E; Supplementary Fig. S4B).

**Figure 4:**
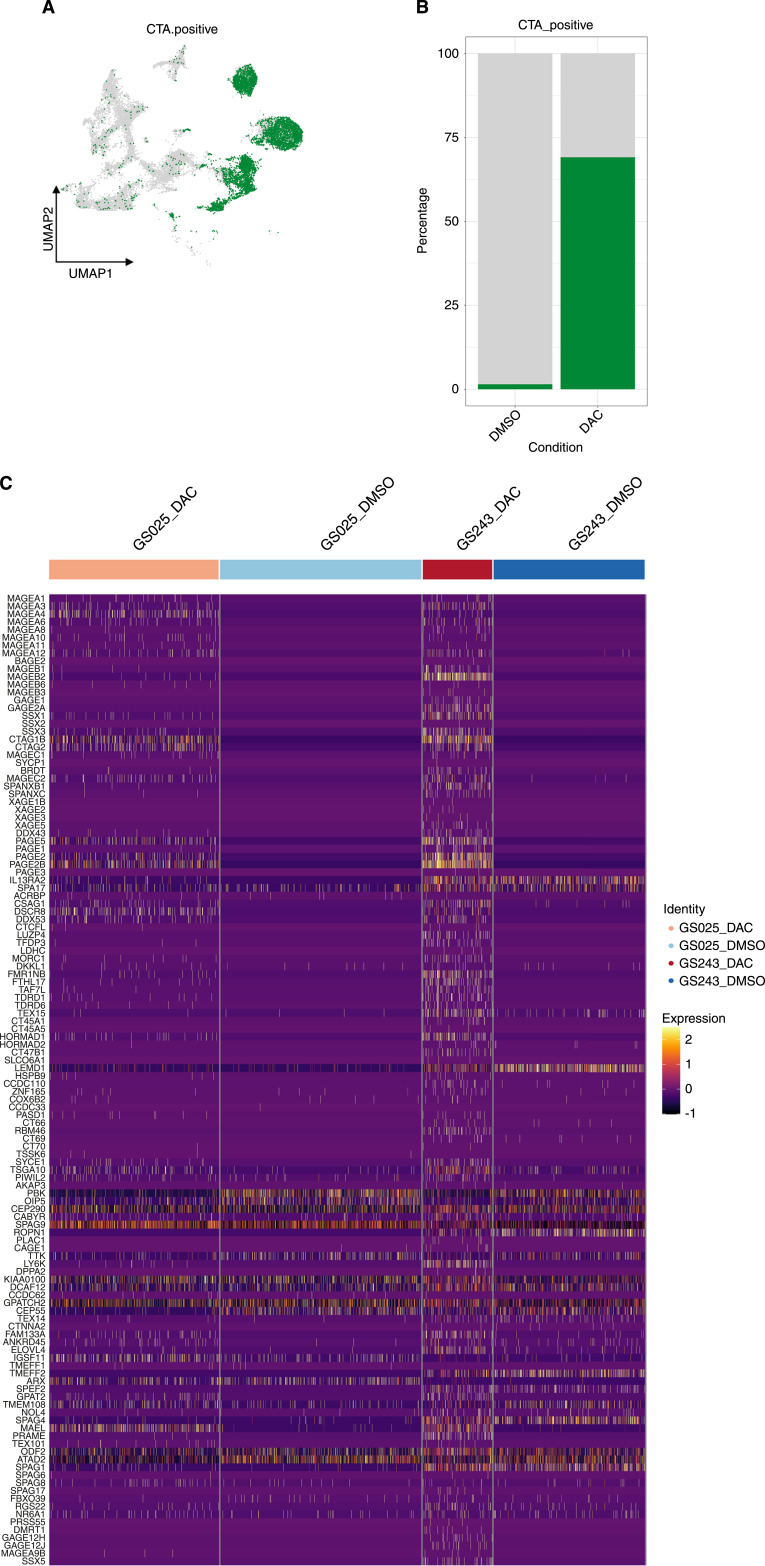
DAC induces multiple CTA for potential polyclonal antigen targeting in gliomaspheres. **A,** UMAP and (**B**) percent of cells with at least one read for seven differentially expressed CTA (*CTAG1B*, *MAGEA4*, *CTAG2* (*LAGE-1*), *PAGE5*, *PAGE2*, *PAGE2B*, and *MAEL*). **C,** Heatmap of CTA expression per cell.

We further assessed the upregulation of other CTA and overall antigen presentation for potential polyclonal antigen targeting. We evaluated the concomitant upregulation of seven differentially expressed CTA (*P*-adj < 0.05). We considered cells to be CTA positive if they contained at least one read from *CTAG1B*, *MAGEA4*, *CTAG2 (LAGE-1)*, *PAGE5*, *PAGE2*, *PAGE2B*, and *MAEL*. About 69.2% of DAC-treated cells compared to 1.5% of DMSO-treated cells were considered to be CTA positive (Fig. 4A and B). While these seven CTA were the most differentially expressed in our scRNA-seq dataset, DAC efficiently induced expression of multiple CTA across our entire single-cell population (Fig. 4C). Moreover, consistent with previous reports of the DAC enhancement of major histocompatibility complex class I (MHC-I) in other cancer types and in glioma (10, 38), DAC-upregulated pan-MHC-I genes (*HLA-A/B/C*) across all cell populations in our dataset (Fig. 3F; Supplementary Fig. S4C). Taken together, these results demonstrate that despite intratumoral and interpatient heterogeneity in patient-derived gliomaspheres, DAC induction of CTA expression was not limited to any specific subpopulations but instead appeared widespread. This widespread CTA upregulation is complimented by enhanced MHC-I antigen presentation, potentially priming glioma to polyclonal antigen targeting.

### DAC induction of CTA but not stimulation of interferon response genes nor MHC-I is correlated with direct CpG demethylation

While analyzing our single cell RNA sequencing dataset, we recognized that inadequate read coverage might limit our detection of DAC-induced CTA and downstream pathway stimulation (39). We therefore validated the transcriptional and epigenetic changes in immunogenicity following DAC treatment of immortalized U-251MG and five patient-derived primary gliomasphere lines (GS025, GS081, GS243, GS277, GS351) with bulk RNA sequencing and probe-based methylation array.

**Figure 5:**
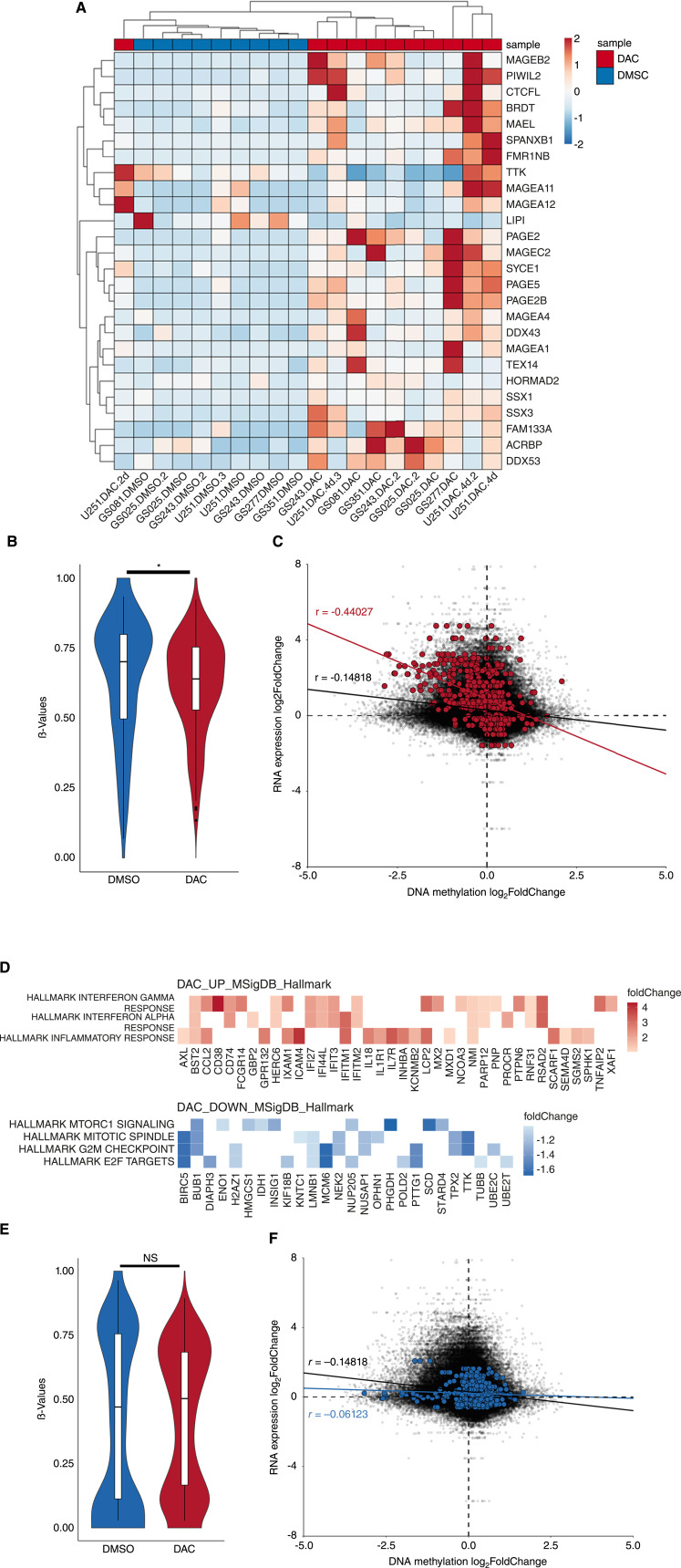
CTA induction but not downstream interferon-downstream stimulation by DAC is correlated with CpG demethylation in glioma cells. **A,** Heatmap and hierarchical clustering of differentially expressed CTA. Scale represents *z*-score of DESeq2 counts. **B,** Violin plot of beta value distribution between treatment conditions for CpG probes corresponding to differentially upregulated CTA (*P*-value = 0.028; Wilcoxon rank sum test with continuity correction). **C,** Scatter plot of all CpG island probes containing intersectional DNA methylation log_2_FoldChange and RNA expression log_2_FoldChange of annotated genes in black (*n* = 115,801 CpG island probes). CpG island probes corresponding to CTA with Pearson correlation are highlighted in (red; *n* = 535 CpG probes; *n* = 65 CTA genes). **D,** Enricher analysis of DESEQ2 differentially upregulated genes (Hallmark IFNγ *P* = 0.00548; Hallmark IFNα *P* = 0.00548; Hallmark inflammatory response *P* = 0.0426) and downregulated genes (Hallmark mTORC1 signaling *P* = 1.59e^−3^; Hallmark mitotic spindle *P* = 1.59e^−3^, Hallmark G2M Checkpoint *P* = 7.77e^−5^; Hallmark E2F targets *P* = 7.77e^−5^). Hallmark MSigDB signature adjusted *P* values determined using Fisher exact test adjusted by Benjamini–Hochberg correction. **E,** Violin plot of beta value distribution between treatment conditions for CpG probes corresponding to differentially upregulated DAC_UP_MSigDB_Hallmark genes (*P*-value = 0.497; Wilcoxon rank sum test with continuity correction). **F,** Scatter plot of intersectional DNA methylation log_2_FoldChange and RNA expression log_2_FoldChange CpG island probes. CpG probes corresponding to the Hallmark Interferon Alpha Response gene set with Pearson correlation are highlighted in blue (*n* = 471 probes; *n* = 53 genes).

We first assessed the overall change in methylation level (β-value). As expected, DAC treatment compared with DMSO vehicle control demonstrated an overall reduction in β-value of 794,441 detected CpG probes in all gliomasphere replicates. In U-251MG, β-value reduction was proportional to time-dependent exposure to DAC (Supplementary Fig. S5A). Within our bulk RNA sequencing dataset, we identified 26 differentially expressed CTA with an adjusted *P*-value less than 0.05. 24 of those CTA had a positive log_2_FoldChange greater than one (Supplementary Fig. S5B). As in our scRNA-seq dataset, we identified robust upregulation of CTA universally in all DAC-treated samples despite biological and interpatient differences in primary cell lines (Fig. 5A).

Within the total methylation dataset containing 794,441 detected CpG probes, 154 CpG probes annotated for the 26 differentially expressed CTA showed significant reduction in average β-value across DAC- and vehicle-treated samples (Fig. 5B). We further subset the total dataset into 490,451 differentially methylated probes containing intersectional data between array and bulk RNA sequencing results and 115,801 of those were annotated within CpG Islands. Filtering on 454 CpG island probes annotated for all 65 published CTA, log_2_FoldChange RNA expression of CTA negatively correlated with log_2_FoldChange DNA methylation of annotated CpG island sites (Fig. 5C). Moreover, for 6 of 12 differentially upregulated CTA we were able to demonstrate significantly demethylated CPG island probes (Supplementary Fig. S5C). Consistent with the epigenetic induction of NY-ESO-1, these results again indicate that DAC induces overall CTA expression via the reversal of DNA hypermethylation in GBM.

To further elucidate global changes resulting from DAC treatment, we performed gene set enrichment analysis on differentially expressed genes with an adjusted *P*-value less than 0.05 using the Hallmark Molecular Signature Database (40). Consistent with our DAC-response populations in our scRNA-seq dataset, DAC significantly upregulated interferon response pathways and general inflammatory response signaling while downregulating cell cycle signatures (Fig. 5D). It is well-characterized that high-dose DAC can induce cell cycle arrest at G2/M phase via formation of DNA adducts in human cancer cells (34). Indeed, even at this low dose exposure, the downregulation of cell cycle signatures translated to delayed proliferation of GBM cells when assayed by xCelligence (Supplementary Fig. S5D). However, the DAC monotherapy control group in previous animal experiments demonstrated no statistically improved survival without TCR-T adoptive administration, indicating that our DAC dosing regimen is not sufficient to induce tumor cell death alone (14).

To better delineate the mechanisms of DAC-induced interferon stimulated genes (ISG) and MHC-I, we again assessed the methylation level of probes associated with differentially upregulated ISG. In contrast to annotated CTA probes, 871 CpG probes annotated for the 75 differentially expressed ISG and 44 CpG probes annotated for *HLA-A/B/C* saw no significant change in average β-value across DAC- and vehicle-treated samples (Fig. 5E; Supplementary Fig. S5E). Correlation analysis of log_2_FoldChange RNA expression and log_2_FoldChange DNA methylation of annotated CpG island sites in the entire MSigDB Hallmark Interferon Alpha Response Gene Set likewise demonstrated no significant negative correlation between ISG CpG island methylation and mRNA expression (Fig. 5F). While 4 of 75 differentially expressed ISG contained at least one significantly demethylated probe, these results indicated that the majority of ISG were not regulated by direct DNA demethylation in our methylation array dataset and we sought to better characterize the mechanisms of their enhancement (Supplementary Fig. S5F).

### The induction of ISG and MHC-I by DAC is associated with hERV reactivation in glioma

**Figure 6:**
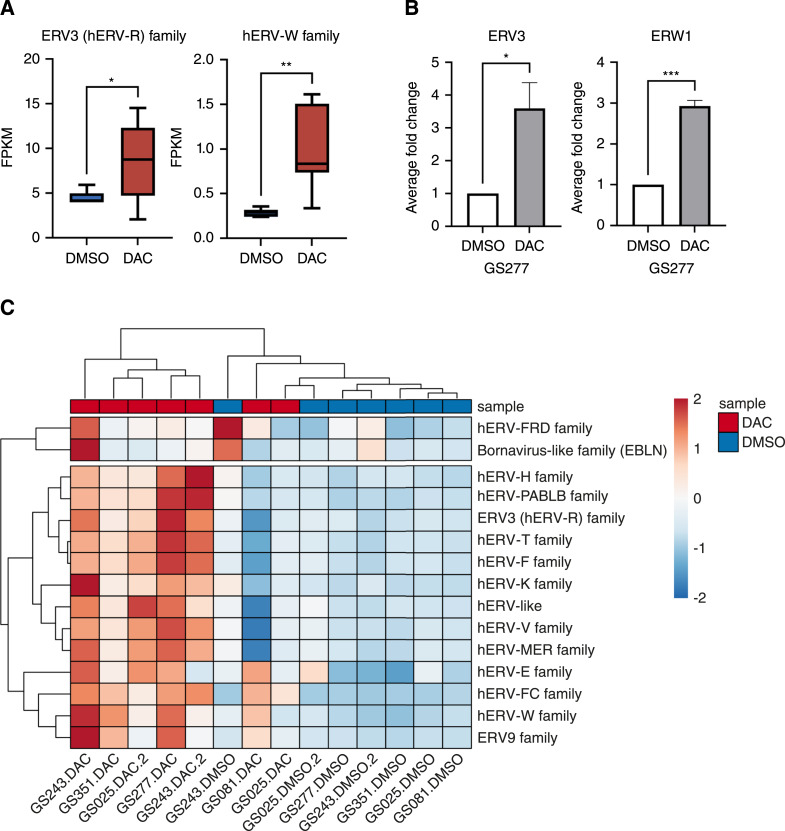
DAC reactivates hERV expression in gliomaspheres. **A,** FPKM of ERV3 and HERVR family for primary gliomasphere cell lines (*n* = 7; *, *P* < 0.05; **, *P* < 0.01; two-tailed unpaired *t* test). **B,** Average fold change of mRNA expression of treated GS277 for *ERV3* and *ERVW1*/Syncytin-1 as measured by qPCR (*n* = 3; *, *P* < 0.05; ***, *P* < 0.001; two-tailed unpaired *t* test). **C,** Heatmap and hierarchical clustering of hERV gene families. Scale represents *z*-score of hERV family FPKM normalized to housekeeping FPKM.

Previous reports have shown that DNMTi trigger type I interferon signaling across a variety of cancer cells by genome-wide DNA demethylation that re-expresses hERV and subsequently activate pattern recognition receptors (PRR) specific for the sensing of viral nucleic acid (41). To better characterize DAC-mediated interferon immune sensitization in GBM cells, we hypothesized that DAC similarly enhances interferon response signaling via the activation of hERV in GBM. Indeed, after aligning our bulk RNA sequencing reads to a synthetic viral metagenome and performing FPKM quantification, we noted the significant upregulation of the ERV3 and hERV-W families (Fig. 6A). Previously attributed to induce robust type I interferon responses, individual hERV genes, *ERV3* (within ERV3 family) and *ERW1* (Syncytin-1, within the hERV-W family), were validated in primary GS277 cells with RT-qPCR (Fig. 6B; ref. 41). Hierarchical clustering of hERV family FPKM normalized to housekeeping FPKM (Supplementary Fig. S6A) likewise demonstrated an overall upregulation of all hERV families contained within the viral metagenome in DAC-treated primary gliomaspheres (Fig. 6C; Supplementary Fig. S6A).

Despite previous reports in other cancer types of hERV type I interferon enhancement via toll-like receptor 3 (*TLR3*) activation, we saw negligible expression of *TLR3* within our scRNA-seq or bulk RNA sequencing datasets (Supplementary Fig. S6B; ref. 41). Indeed, the prevalence of *TLR3* expression in gliomas is poorly understood (42), but we noted the concomitant expression of other PRR: retinoic acid-inducible gene I (RIG-I/*DDX58*), melanoma differentiation-associated protein 5 (MDA-5/*IFIH1*), stimulator of interferon genes (*STING1*), and mitochondrial antiviral-signaling protein (*MAVS*; Supplementary Fig. S6C–S6F). Previous studies have implicated the activation of RIG-I, RIG-I like receptors, and STING pathways by hERV for type I interferon activation and we hypothesized that DAC upregulates ISG via the demethylation of hERV and subsequent PRR activation in GBM (43).

## Discussion

The induction of CTA using DAC for targeting by the immune system has shown increasing success in numerous solid tumor sites (4, 7). Indeed, there are more than 150 registered clinical trials in various states of completion involving the use of NY-ESO-1 alone with several using DAC as an immunosensitizing agent (5, 6). In this study, we bridged several key gaps in the administration of DAC to induce tumor-associated antigens and immunogenic enhancement in GBM. We confirmed that NY-ESO-1, a representative CTA, is downregulated by CpG hypermethylation in the majority of GBM. As such, we demonstrated that the reversal of this DNA hypermethylation profile using DAC-induced NY-ESO-1 antigen for targeting and polyfunctional enhancement of NY-ESO-1 engineered TCR-T cells. Methylomic and single-cell transcriptomic profiling further demonstrated that DAC similarly demethylated and induced other tumor-associated CTA for potential polyclonal adoptive T-cell and cancer vaccine therapies. Finally, DAC-reactivated hERV expression that was associated with interferon signaling to successfully prime GBM cells for enhanced immunotherapeutic targeting.

The use of DAC as an immunosensitizing agent is particularly attractive for the treatment of GBM. This study and others found that the key antigens frequently absent in brain tumors can be induced epigenetically for immune targeting (10–14). However, the immunosuppressive tumor microenvironment and limited effector activation and infiltration into the tumor remain a major challenge for current immunological therapies for the treatment of GBM (2, 3).

Promisingly, previous studies have demonstrated that the inhibition of DNA methylation by DNMTi has substantial benefit in addition to the induction of tumor-associated antigens. Administration of DNMTi in solid tumors has been shown to stimulate antiviral interferon signaling through the demethylation of hERV, resulting in enhanced sensitivity to immune checkpoint therapy (41). In tumor-reactive effector cells, *de novo* hypermethylation programs have been associated with exhausted T-cell phenotypes (44, 45).

Our experiments did not directly characterize DAC-mediated effects in the GBM tumor-immune microenvironment. While we have previously reported adoptive NY-ESO-1 TCR-T trafficking and antigen specific cytolysis in an orthotopic xenograft model, future *in vivo* experiments in an immunocompetent model will need to be performed to delineate the mechanisms of DAC-mediated hERV stimulation and downstream interferon activation (14).

Furthermore, the exact mechanisms by which DAC might enhance hERV, ISG, and MHC-I expression in glioma remains to be determined. While our transcriptomic data of serum-free patient-derived glioma cell lines suggest an activation of these antiviral-mediated inflammatory signaling and MHC-I after DAC treatment in glioma, future experiments will need to mechanistically establish the specific hERV upregulation and downstream PRR activation in glioma. Indeed, the study of hERV-activated pathways remains complex and the exact mechanisms by which DAC might induce these pathways in glioma requires future investigation (43). We speculate that this immunogenic enhancement may remodel the tumor-immune microenvironment in glioma to further attract, stimulate, and amplify antitumor effector cell responses.

In conclusion, we demonstrated that reversal of hypermethylation in CTA primed glioma to polyfunctional and polyclonal TCR-T effector cell targeting. Synergistically, DAC-reactivated hERV and upregulated key interferon signaling pathways that augments immune cell function against glioma. Combined with targeted, robust, and polyfunctional TCR-T effector cell responses, our data strongly support a promising strategy for the treatment of GBM using DAC to epigenetically prime tumor cells for immunotherapeutic targeting.

## Supplementary Material

Supplementary Data 1 Isoplexis fluorescence values per cytokine per cell

Supplementary Data 2 hERV raw counts and FPKM values

Supplementary Figure 1 Fig S1A-D

Supplementary Figure 2 Fig S2 A-C

Supplementary Figure 3 Fig S3 A-C

Supplementary Figure 4 Fig S4 A-C

Supplementary Figure 5 Fig S5 A-F

Supplementary Figure 6 Fig S6 A-F
