# Supplementary material for: Epigenetic Induction of Cancer-Testis Antigens and Endogenous Retroviruses at Single-Cell Level Enhances Immune Recognition and Response in Glioma
Source: Cancer Res Commun. 2024 Jul 26;4(7):1834–49. doi: 10.1158/2767-9764.CRC-23-0566 (PMC11275559; doi:10.1158/2767-9764.CRC-23-0566)
Supplement: Supplementary Figure 1 — Fig S1A-D [file crc-23-0566_supplementary_figure_1_supp1.pdf]

Figure S1

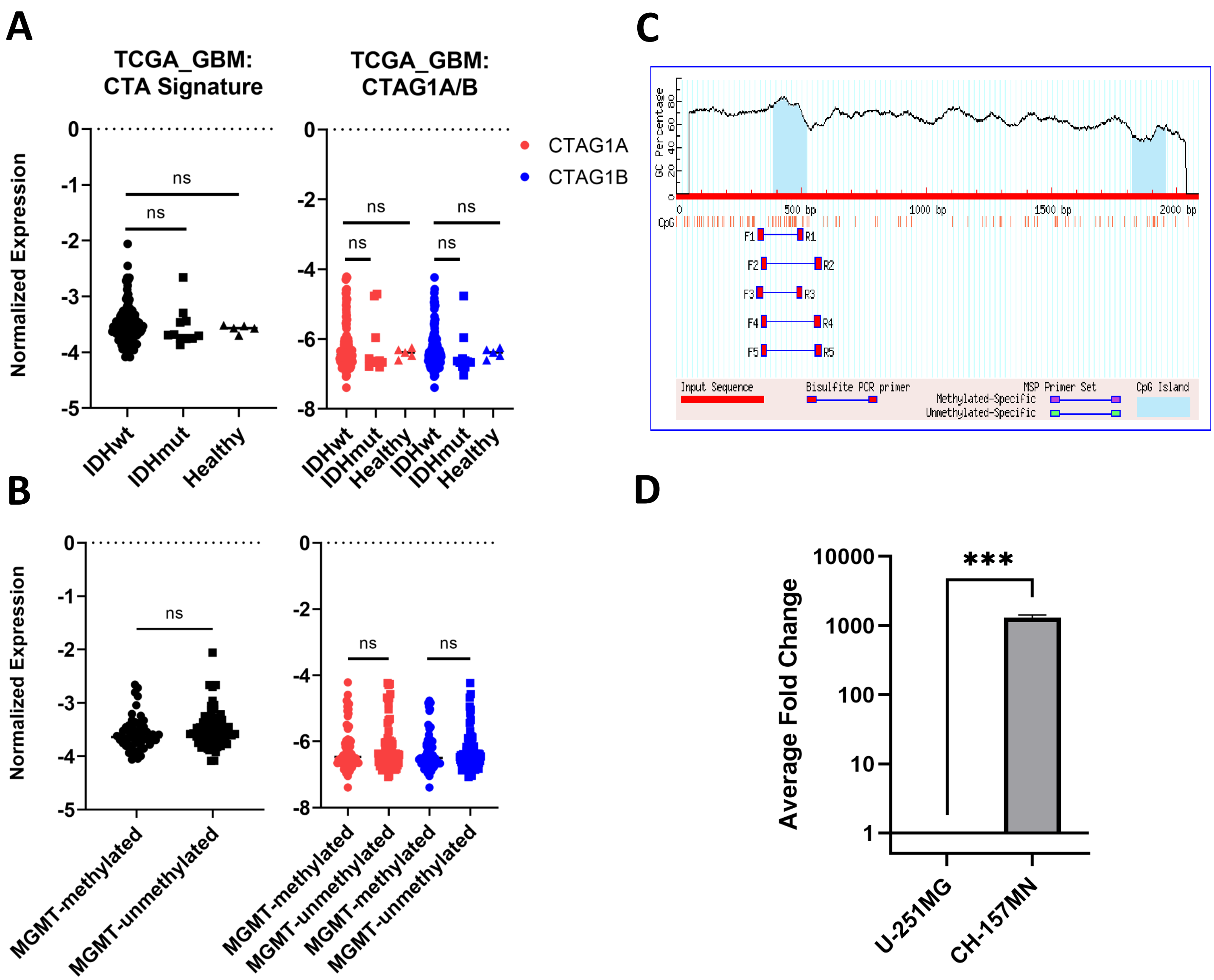

Fig. S1: (A-B) Normalized expression of CTA Signature, CTAG1A, and CTAG1B in TCGA_GBM samples subset into (A) IDHmut (n = 11), IDHwt (n = 142), and healthy brain samples (n = 5) or **(B)** MGMT-methylated (n = 52) and MGMT-unmethylated (n = 72) (ns = not significant, two-sample t test). **(C)** CpG Island identification and Bisulfite PCR primer locations. **(D)** CTAG1B expression in CH-157MN measured by RT-qPCR (n = 3, ***P < 0.001, two-tailed unpaired t test).
