# Supplementary material for: Epigenetic Induction of Cancer-Testis Antigens and Endogenous Retroviruses at Single-Cell Level Enhances Immune Recognition and Response in Glioma
Source: Cancer Res Commun. 2024 Jul 26;4(7):1834–49. doi: 10.1158/2767-9764.CRC-23-0566 (PMC11275559; doi:10.1158/2767-9764.CRC-23-0566)
Supplement: Supplementary Figure 2 — Fig S2 A-C [file crc-23-0566_supplementary_figure_2_supp2.pdf]

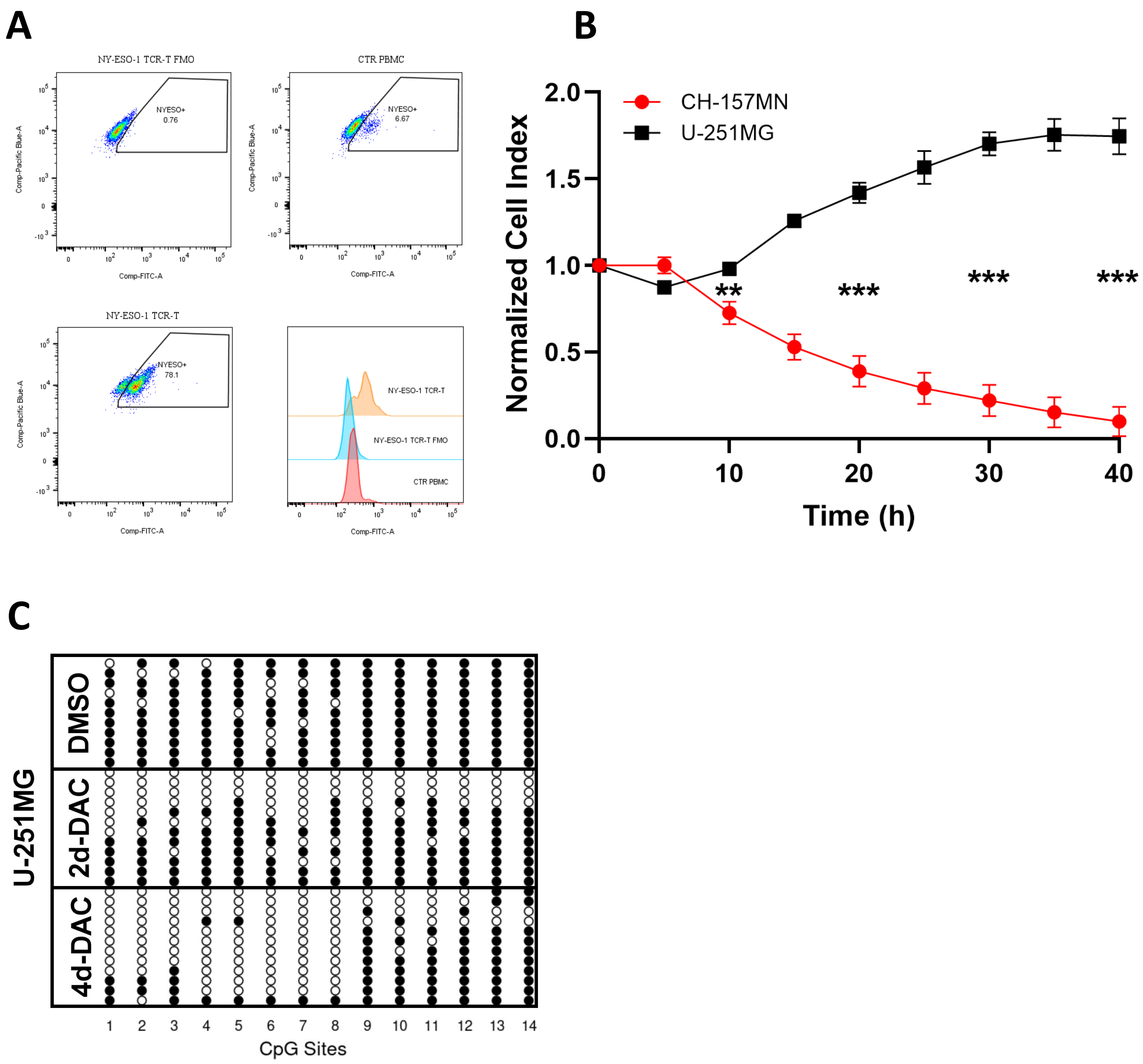

Fig. S2: (A) Representative flow cytometry plots demonstrate >75% NY-ESO-1 TCR transduction (Comp FITC-A) in CD8+ cells (Comp-Pacific Blue-A) compared to 6% in control untransduced CD8+ cells. **(B)** Representative normalized cell index output of real-time impedance-based xCelligence assay (n = 2, **P < 0.01, ***P < 0.001, ****P < 0.0001, unpaired t test). **(C)** Representative Bis-Seq-TA cloning lollipop diagram of all 14 analyzed CpG sites in U-251MG.
