# Supplementary material for: Epigenetic Induction of Cancer-Testis Antigens and Endogenous Retroviruses at Single-Cell Level Enhances Immune Recognition and Response in Glioma
Source: Cancer Res Commun. 2024 Jul 26;4(7):1834–49. doi: 10.1158/2767-9764.CRC-23-0566 (PMC11275559; doi:10.1158/2767-9764.CRC-23-0566)
Supplement: Supplementary Figure 3 — Fig S3 A-C [file crc-23-0566_supplementary_figure_3_supp3.pdf]

Figure S3

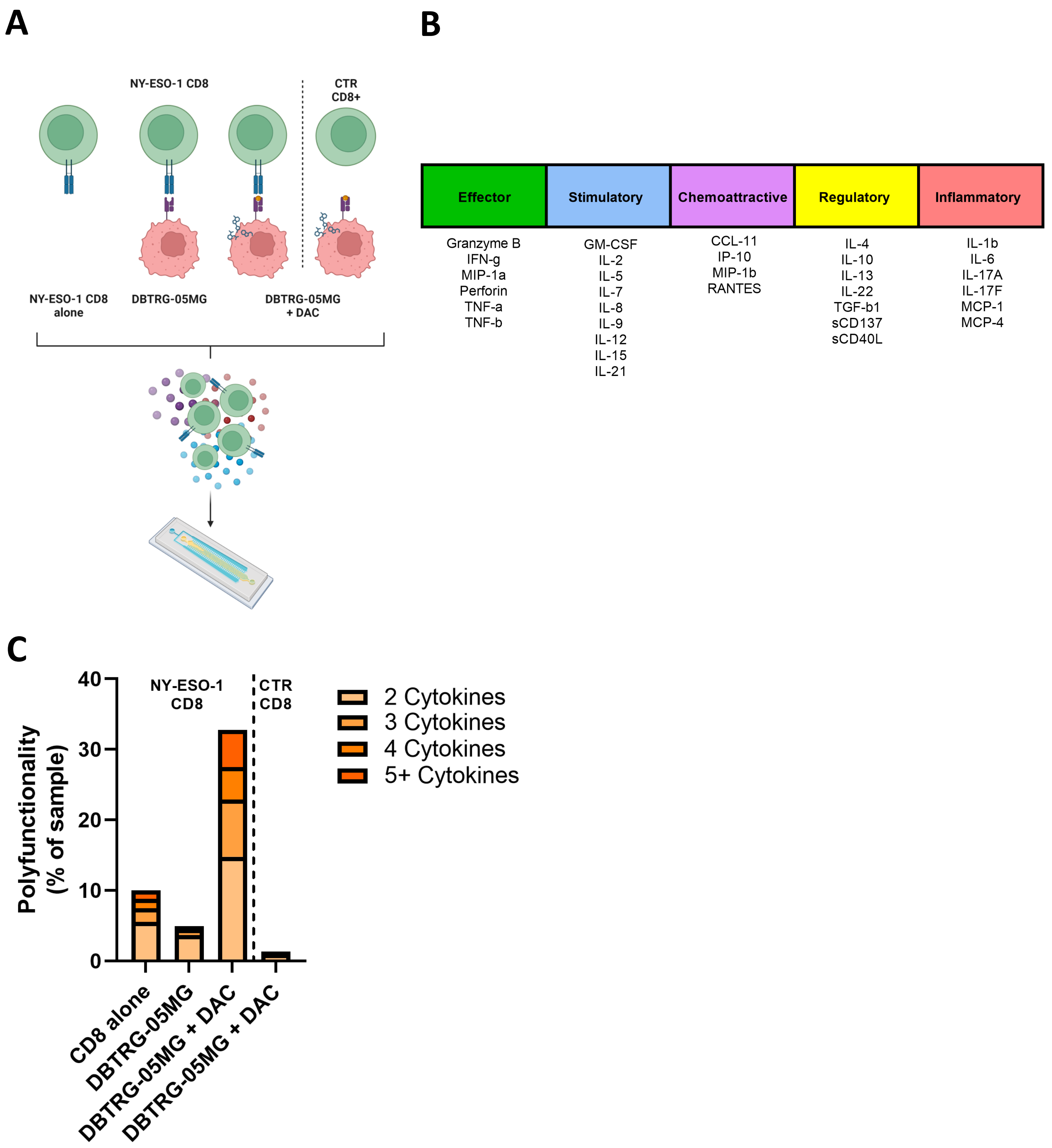

Fig. S3: (A) NY-ESO-1 TCR CD8+ and untransduced control CD8+ were co-cultured for 20 hours with DBTRG-05MG pre-treated with DAC. Following co-culture, CD8+ were loaded into IsoPlexis single cell secretomics assay. Created with BioRender.com. **(B)** A panel of 32 key immunologically relevant molecules across major categories (Effector, Stimulatory, Chemoattractive, Regulatory, and Inflammatory) assayed for immune cell polyfunction. **(C)** NY-ESO-1 CD8+ effector cell targeting of DAC-treated cells results in polyfunctional (2+) cytokine release.
