# Supplementary figures and images for: Epigenetic Induction of Cancer-Testis Antigens and Endogenous Retroviruses at Single-Cell Level Enhances Immune Recognition and Response in Glioma
Source: Cancer Res Commun. 2024 Jul 26;4(7):1834–49. doi: 10.1158/2767-9764.CRC-23-0566 (PMC11275559; doi:10.1158/2767-9764.CRC-23-0566)

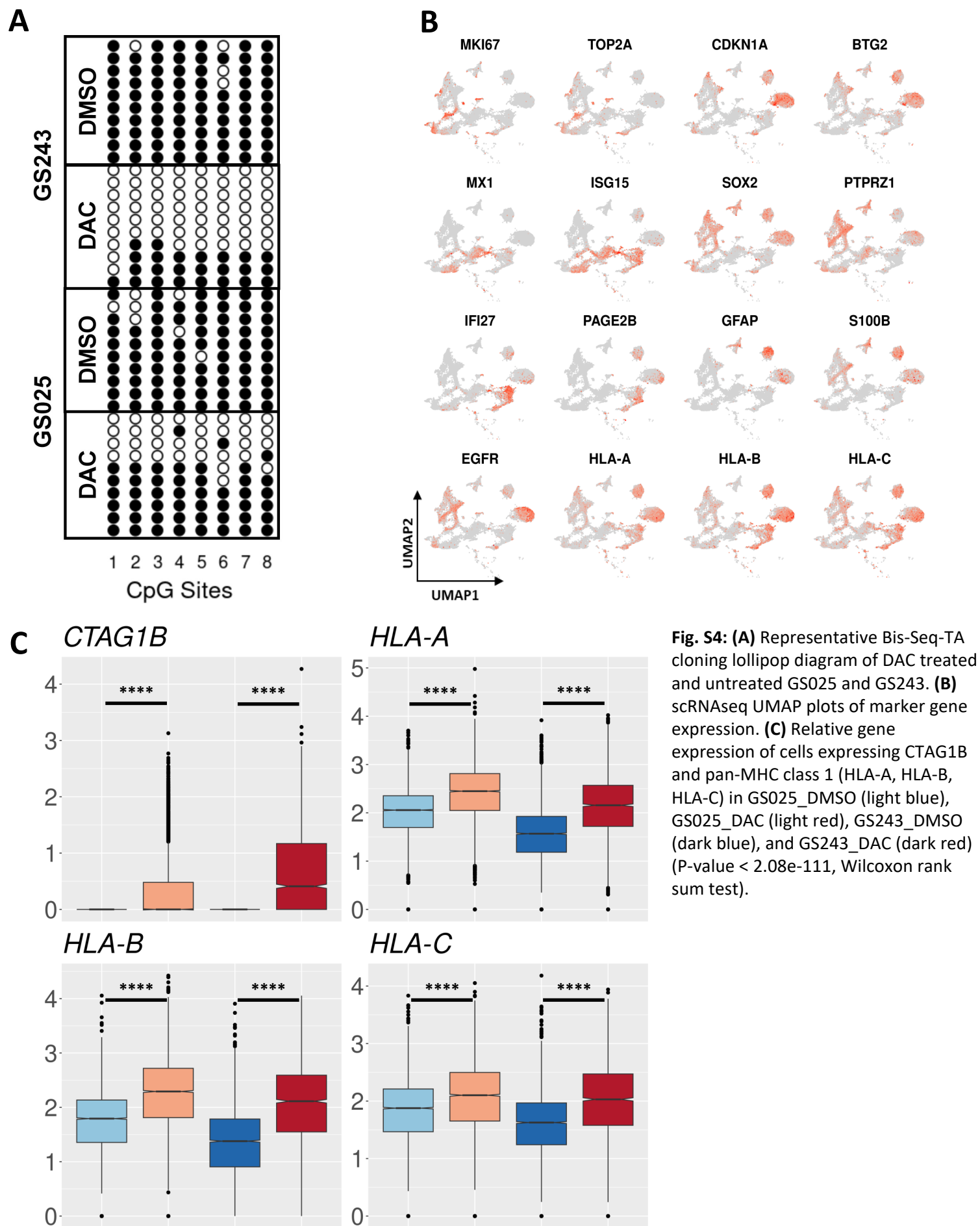

Supplement: Supplementary Figure 4 — Fig S4 A-C [file crc-23-0566_supplementary_figure_4_supp4.pdf]
