# Supplementary material for: Epigenetic Induction of Cancer-Testis Antigens and Endogenous Retroviruses at Single-Cell Level Enhances Immune Recognition and Response in Glioma
Source: Cancer Res Commun. 2024 Jul 26;4(7):1834–49. doi: 10.1158/2767-9764.CRC-23-0566 (PMC11275559; doi:10.1158/2767-9764.CRC-23-0566)
Supplement: Supplementary Figure 5 — Fig S5 A-F [file crc-23-0566_supplementary_figure_5_supp5.pdf]

Figure S5

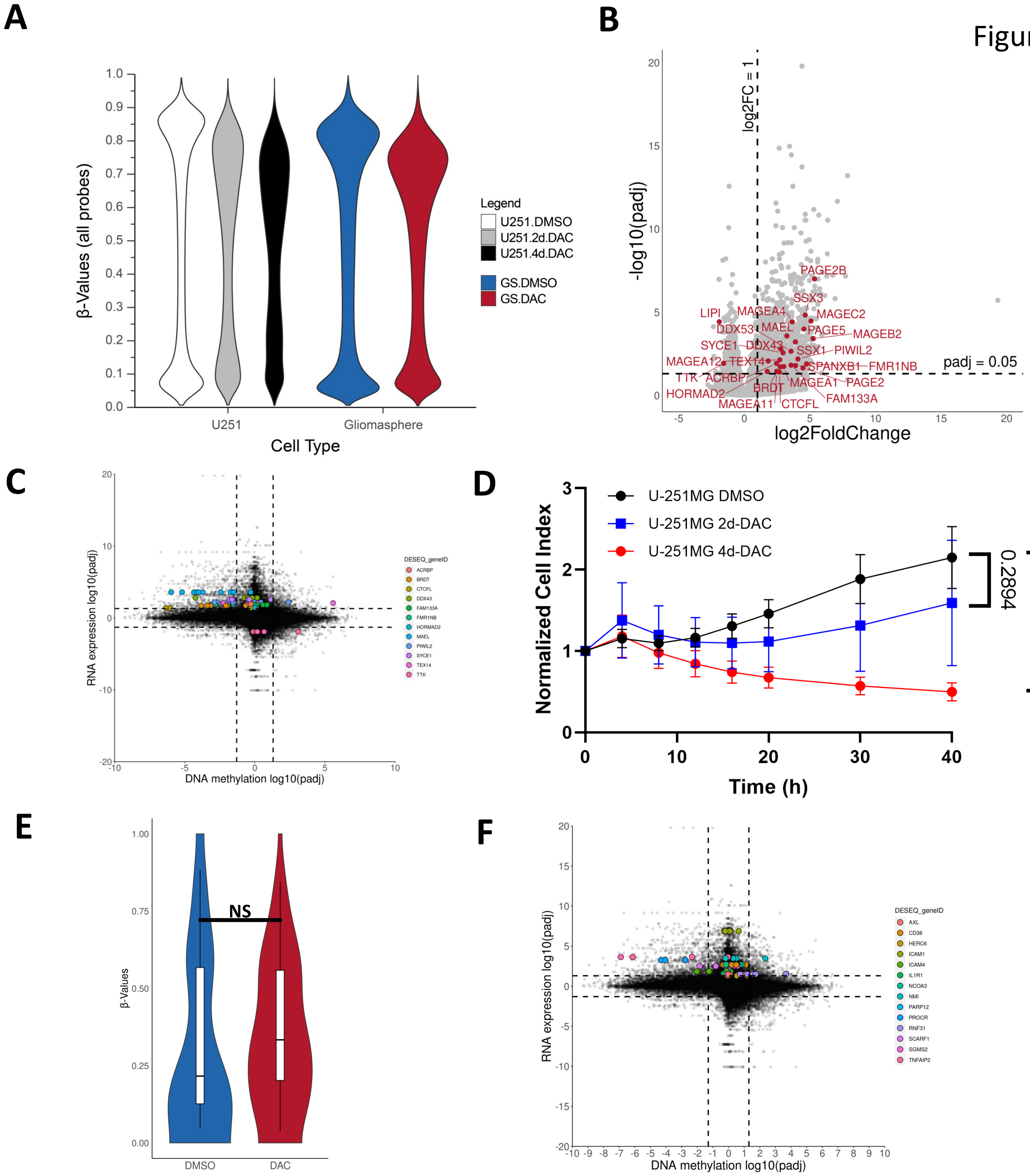

Fig. S5: (A) Violin plot of raw beta value distribution across treatment timepoint in U-251MG (P-value = 2.2e-16, Wilcoxon rank sum test with continuity correction) and across all primary gliomasphere replicates (P-value = 2.2e-16, Wilcoxon rank sum test with continuity correction). **(B)** Volcano plot of differentially expressed CTA with an adjusted p-value (padj) less than 0.05 and a log 2 fold change (log2FC) greater than 1 as computed by DESEQ2. **(C)** Starburst plot for comparison of differentially methylated CpG island probes and differential RNA expression for each CTA gene. Dotted lines represent P-adj = 0.05. If a mean DNA methylation β-value or mean gene expression value is higher (greater than zero) −1 is multiplied to log10(padj), providing positive values. CpG island probes corresponding to differentially expressed CTA are highlighted (n = 83 probes, n = 12 CTA). **(D)** Representative normalized cell index output of real-time impedance-based xCelligence assay (n = 3, unpaired t test). **(E)** Violin plot of beta value distribution between treatment conditions for CpG probes corresponding to HLA-A/B/C genes (P-value = 0.292, Wilcoxon rank sum test with continuity correction). **(F)** Starburst plot of CpG island probes corresponding to differentially expressed IFN response genes are highlighted (n = 77 probes, n = 14 genes).
