# Supplementary material for: Epigenetic Induction of Cancer-Testis Antigens and Endogenous Retroviruses at Single-Cell Level Enhances Immune Recognition and Response in Glioma
Source: Cancer Res Commun. 2024 Jul 26;4(7):1834–49. doi: 10.1158/2767-9764.CRC-23-0566 (PMC11275559; doi:10.1158/2767-9764.CRC-23-0566)
Supplement: Supplementary Figure 6 — Fig S6 A-F [file crc-23-0566_supplementary_figure_6_supp6.pdf]

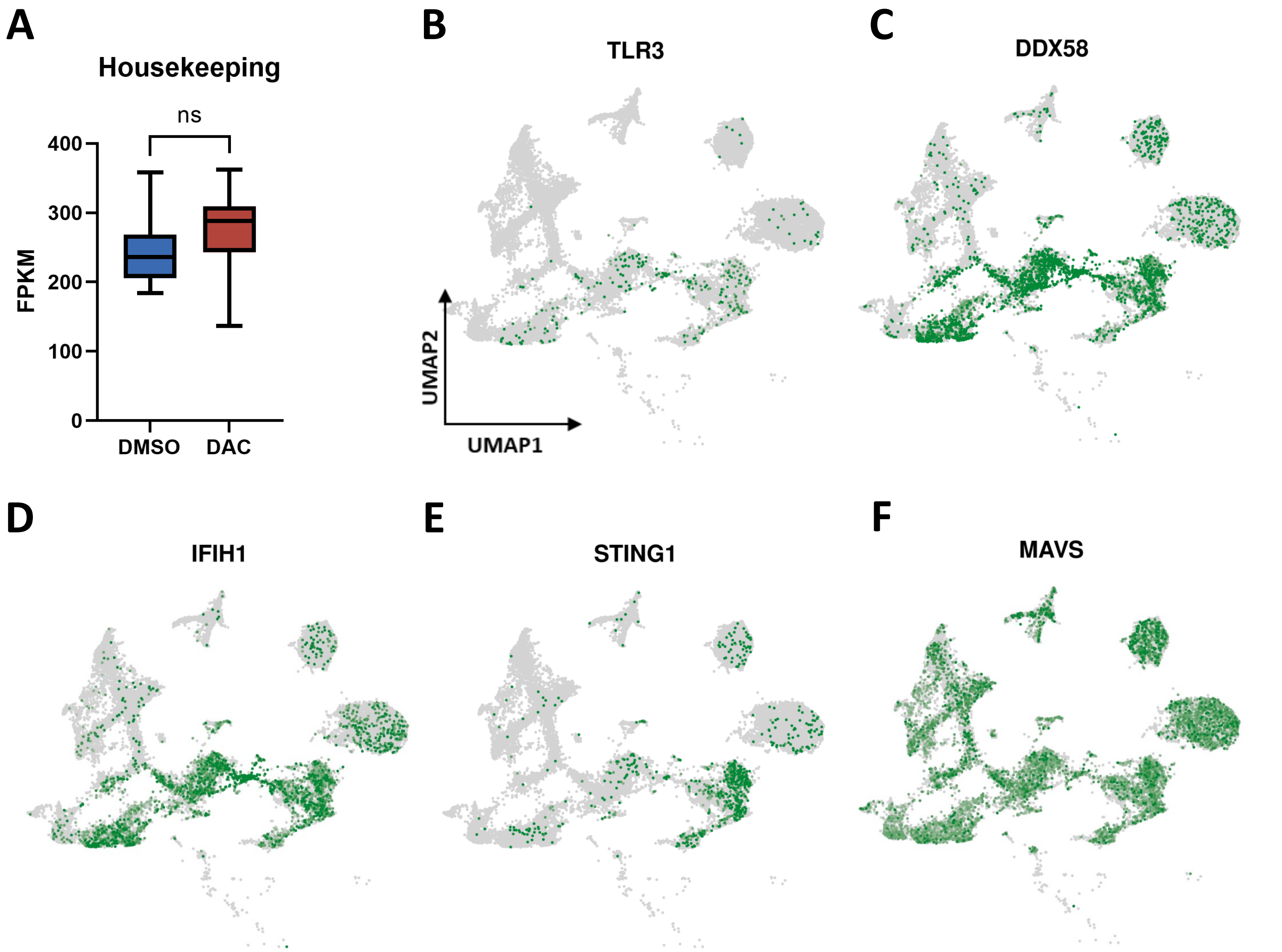

Fig. S6: (A) FPKM quantification of primary GS bulk RNA sequencing reads aligned to housekeeping gene sequences in viral metagenome. **(B-F)** UMAP of cells expressing pattern recognition receptors **(B)** TLR3, **(C)** RIG-I (DDX58), **(D)** MDA5 (IFIH1), **(E)** STING1, and **(F)** MAVS.
